# Simultaneous Detection of Collagen I Alpha II and Cytokeratin 19 mRNA by Multiplex qPCR in Liquid Biopsy in Diagnosis of Patients with Resectable Solid Tumors

**DOI:** 10.3390/ijms25179567

**Published:** 2024-09-03

**Authors:** Lara Sofía Estévez Pérez, Begoña O. Alén, María Otero Alén, Saioa Domínguez Hormaetxe, Laureano Simón, Ángel Concha

**Affiliations:** 1 Pathology Department, Biomedical Research Institute A Coruña (INIBIC), University Hospital Complex A Coruña, 15006 A Coruña, Spain; angel.concha.lopez@sergas.es; 2 Santiago de Compostela Health Research Institute (IDIS), University Hospital Complex Santiago de Compostela, 15706 Santiago de Compostela, Spain; maria.otero.alen@sergas.es; 3 Oncomatryx Biopharma, 48160 Derio, Spain; sdominguez@oncomatryx.com (S.D.H.); lsimon@oncomatryx.com (L.S.)

**Keywords:** COL1A2, KRT19, early detection multi-cancer screening, multiplex RT-qPCR, liquid biopsy

## Abstract

The early detection of tumors is one of the key factors in increasing overall survival in cancer patients. A wide range of cancers still do not have a system of early diagnosis; therefore, the development of new non-invasive tools in this line is essential. Accordingly, the objective of our work was to develop a non-invasive screening method for the early detection of various carcinomas in plasma using a panel that combines two markers using RT-qPCR. A retrospective case-control study was conducted to develop a cancer screening test based on the detection of stromal and epithelial biomarkers (COL1A2 and KRT19) in plasma. The expression of biomarkers was evaluated using multiplex quantitative PCR applied to 47 cases with non-metastatic tumors and 13 control participants. For both biomarkers, a cut-off value was stablished using Youden’s J index through ROC curve analysis and areas under the curve (AUC) were calculated. The plasma mRNA expression level of both biomarkers was significantly higher in diseased versus healthy patients. Moreover, ROC curve analysis showed an AUC value of 0.897 for the combined model. This model also resulted in a cutoff value of 0.664, as well as a sensitivity of 83% and a specificity of 84.6%. These results suggest that the plasma expression levels of COL1A2 and KRT19 could a have potential role in detecting various types of cancer at the early stages. The combined analysis of both stromal and epithelial biomarkers would provide a non-invasive screening method that would allow us to differentiate patients with an active neoplastic process.

## 1. Introduction

Despite the advances in surgical techniques and targeted treatments in recent years, cancer remains one of the leading causes of death in the world [1]. Early detection is one of the key factors in increasing oncological overall survival. When cancer is detected in the early stages, treatment is more effective, and survival is dramatically improved. However, around 50% of cancers are detected at an advanced phase [2,3]. Currently, various screening systems are implemented in clinical practice to select the population susceptible to undergoing curative surgery, but these methods have been designed for specific tumors, and high false-positive rates lead to invasive interventions for definitive diagnosis [4,5]. There are various issues and different aspects of the approach that we face when talking about early detection. However, certainly, two of these essential issues are the search for and validation of biomarkers, and the development or improvement of new detection technologies [6]. In this regard, liquid biopsy plays a fundamental role, since it is a non-invasive technique that can be used as a screening/early detection method to monitor responses to treatment and detect minimal residual disease or early recurrence. This would mean we are able to offer, in addition, molecular information at each moment of disease evolution [7,8]. New multi-cancer blood detection tests at resectable stages have been published in recent years, thus favoring the early detection of the tumor process [9,10,11], but further validation is needed before they can be incorporated into cancer screening programs. The development of new screening tools is essential to detect the most frequent and high-mortality cancers in order to adopt a curative therapeutic strategy when they are still amenable to treatment. Moreover, in addition to the importance of early diagnosis, these new tools are also crucial to better understand the tumor mechanisms involved in tumor progression that can be used to explore new treatment strategies. Carcinomas are the most frequent malignant tumors in the adult population [1]. They are constituted by epithelial cells, which are the neoplastic proliferating elements in the strict sense, and by the tumor stroma, which is fundamentally represented by specialized fibroblasts (CAFs), blood vessels, and inflammatory cells [12]. Cytokeratins are characteristic intermediate filaments of the cytoskeleton of epithelial cells, although they might occasionally be detected in other cell lineages [13]. However, of the 20 types characterized, cytokeratin 19 (KRT19), which is a low-molecular-weight type I cytokeratin, is the one with the most specific epithelial character, so its identification is used in the detection of macro-/micrometastases in lymph nodes [14]. On the other hand, CAFs are highly specialized fibroblasts that play a crucial role in the initiation and progression of cancer and possess a distinctive phenotype, characterized by the expression of molecules such as fibroblast-associated protein (FAP), collagen XI alpha I (COL11A1) or collagen I alpha II (COL1A2) [15]. In a previous study, our group concluded that the detection of COL1A2 and KRT19 mRNA in the extracellular vesicles (EVs) of patients with advanced malignant solid tumors could be a valid and reliable neoplastic detection method [16]. In the present work, the aim of our research was to demonstrate that the simultaneous detection of both markers using a multiplex assay is also a useful diagnostic method in patients with resectable non-metastatic carcinomas in the early stages.

## 2. Results

### 2.1. Preparation of the Standard Curve

Universal human reference RNA [XpressRef Universal Total RNA (1 μg/uL), QIAGEN, Valencia, CA, USA] comprising total RNA from 20 different normal human adult and fetal major organs was used for multiplex RT-qPCR development. Standard curves were constructed from 10-fold serial dilutions (10^−1^ to 10^−7^ 10−1 to 10−7, 100 to 1–4 ng/uL) and assayed by RT-qPCR in triplicates.

### 2.2. Simplex RT-qPCR Standardization 

Before multiplexing, all targeted gene primer/probe sets and RT-qPCR reagents were tested using simplex RT-qPCR. Universal human reference RNA was used for RT-qPCR development. The thermocycler was calibrated to obtain the best fluorescent performance. Standard curves were constructed from 10-fold serial dilutions, and the simplex reactions were repeated at least three times for COL1A2 and KRT19 genes and one IC gene actine (ACTB). The average cycle threshold (Ct) values with standard deviations (SDs) are portrayed in Table S1.

### 2.3. Repeatability and Reproducibility of Intra- and Inter-Assay of RT-qPCR

The repeatability and reproducibility of the assay was determined by testing the standard curve. The intra-assay test was performed in quintuplicate within the same run, and the inter-assay test was conducted independently as five different runs in three different days. The coefficients of variation (CVs) for the Ct values of the intra- and inter-assay comparisons were determined (Table 1). From 10^−1^ to 10^−7^ dilution, the values of SD and CV intra-assays ranged from 0.11 to 1.15 and 0.63% to 3% respectively. On the other hand, the values of the SD and CV inter-assays ranged from 0.23 to 0.95 and 0.74 to 3.52%, respectively. Therefore, the variability test for all investigated transcripts was low in the inter-test experiments (<3.52%) and even lower in the intra-test experiments (<3.00%). These data indicated that the assay was repeatable and reproducible with low variation. 

### 2.4. Absence of Interactions between Real-Time Reactions

**Figure 1:**
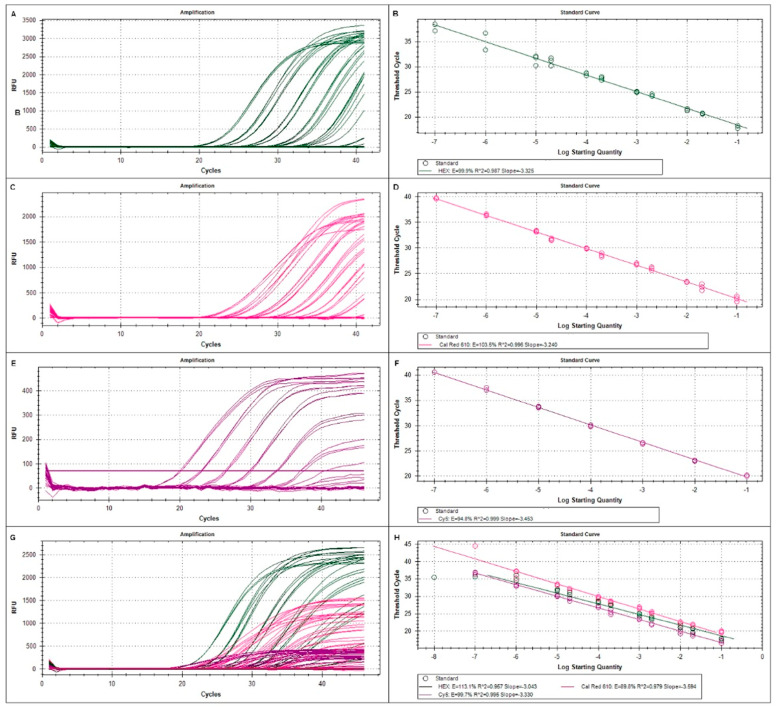
RT-qPCR amplification plots of using seven different 10-fold dilutions (10^−1^ to 10^−7^) of standard RNA in singleplex and multiplex reactions. For COL1A2 (**A**,**B**), KRT19 (**C**,**D**) and ACTB (**E**,**F**) four intermedia points were added. The assay was performed as a singleplex and multiplex reactions to investigate the potential interaction between the primers. Overlapping amplification curves in the singleplex (**A**,**C**,**E**) and triplex reactions (**G**,**H**) did not identify any inhibitory effect. Ct values were plotted on the y axis and serial ten-fold dilutions of the RNA template on the x axis (right panel).

To verify that there were no committed interactions between the three target genes of the multiplex, we also conducted singleplex and triplex real-time reactions with all three targets (ACTB, COL1A2, and KRT19) using 10-fold dilutions of standard RNA. The assay was performed to investigate the potential interaction between the primers. We could observe that the amplification curves for each gene from the singleplex and triplex reactions overlapped (Figure 1, left panel), confirming that the simultaneous amplification of all three targets did not limit the amplification of any individual marker. Typical Ct values fell between 17 and 36 cycles.

### 2.5. Limit of Detection (LOD) and RT-qPCR Efficiency

Standard RNA dilutions ranging from 10^−1^ to 10^−7^ were also prepared to detect the LOD for COL1A2 and KRT19 biomarkers. The amplification plots, amplification efficiencies (E), R2 scores, and slope values are represented in Figure 1 and Table 2. The most sensitive LOD of the assay with triplicate samples was 0.002 ng (0.0001 ng/uL, Table 1). The E-value of the COL1A2 and KRT19 genes was determined to be 99.9% and 103.5%, respectively, in the simplex assay, associated with R2 values of 0.996 (COL1A2) and 0.999 (KRT19). In the multiplex assay, the efficiency for COL1A2 was 113.1%, and for KRT19, it was 89.8%. The R2 score value was 0.957 for COL1A2 and 0.979 for KRT19 (Figure 1). The reaction efficiencies calculated using the standard curves were close to 100% for all three genes, and there were no substantial differences between singleplex and multiplex reactions.

### 2.6. Baseline Characteristics of Patients

Thirteen healthy donors as asymptomatic controls (ACs) and forty-seven cancer patients (CPs) were included in this study. All of them underwent blood extraction for healthcare purposes, and samples were analyzed in the Pathology Department at the University Hospital Complex of A Coruña. Clinical data such as gender, age, tumor histology, and disease stage were obtained from medical records and are summarized in Table 3. 

Regarding the control group corresponding to healthy patients, 13 individuals aged between 26 and 58 years who had not previously presented any oncological process were analyzed. Patients also had no history of chronic pathologies. Among these 13 patients, 30.77% were men (n = 4) and 69.23% were women (n = 9).

In the group of CPs, we analyzed plasma samples of 31 colon adenocarcinomas (CRCs), six renal cell carcinomas (RCs), four bladder cancers (BCs), three prostate adenocarcinomas (PCs) and a small group of others who included one renal leiomyosarcoma, one ductal breast carcinoma and a penile cancer. All of them underwent curative resection. Among these 47 patients, 48.94% were men (n = 23) and 51.06% were women (n = 24). Most of them were in a very early stage of the disease at the time of analysis, and none of them presented local or distant metastases (pTxN0M0). The staging of the different tumors was evaluated according to the eighth edition of the TNM classification of the American Joint Committee on Cancer of 2017 [17].

### 2.7. Comparative Analysis of mRNA Expression Level in Extracellular Vesicles of ACs vs. CPs

**Figure 2:**
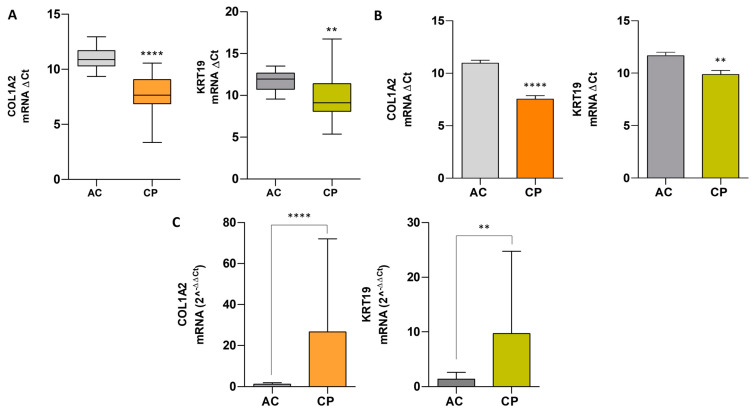
Comparative analysis of mRNA expression level in plasma of ACs vs. CPs. (**A**) Box plot showing the distribution of ΔCt values for tumoral biomarkers (COL1A2 and KRT19) in ACs (n = 13) and CPs (n = 47). The median ΔCt value is represented as a black line within the box plot, and it divides the ΔCt values into lower and upper quartile ranges. The whiskers represent the upper and lower data range in AC and CP samples. Data showed significant differences in ΔCt values between two cohorts of patients (*p* < 0.0001 for COL1A2 and *p* = 0.0031 for KRT19). (**B**) Comparison of ΔCt values of COL1A2 and KRT19. Statistic tests showed significant different expressions between groups in both biomarkers (COL1A2, *p*-value < 0.0001, KRT19, *p*-value < 0.0040). (**C**) Relative fold expression of COL1A2 and KRT19 between AC and CP cohorts of patients. Data showed higher expression levels of COL1A2 and KRT19 in CP individuals. Mann–Whitney U tests showed that these differences were significant between groups in all biomarkers (COL1A2, *p* < 0.0001 and KRT19, *p* = 0.0031). Data are expressed as mean ± SD. Asterisks denote significant differences between groups ACs and CPs for each gene (**** *p*-value < 0.0001; ** *p*-value < 0.01).

The differences between the mean target-gene Ct and reference-gene Ct were denoted as ΔCt. A box-plot diagram was constructed to visualize the distributions of ΔCt in ACs and CPs, as shown in Figure 2A. The distribution of the data showed that the median ΔCt for COL1A2 in ACs and CPs was 10.88 (IQR: 10.31–11.70) and 7.64 (IQR: 6.87–10.56), respectively. The median ΔCt for KRT19 in ACs was 10.95 (IQR 10.73–12.66), and for CPs it was 9.12 (IQR 8.10–11.40). On the other hand, in COL1A2, the means ± SD yielded a value of 10.96 ± 0.98 and 7.53 ± 1.85 for ACs and CPs. For KRT19, the means ± SD yielded a value of 11.66 ± 1.22 and 9.87 ± 2.49 for ACs and CPs, respectively (Figure 2B). The relative gene expression level was evaluated using a modified comparative Ct method, (2−ΔΔCt), as described previously by Pfaffl (17) for each biomarker. Figure 2C shows expression fold changes in the AC and CP samples. COL1A2 showed an increase expression in CPs (9.68 ± 15.07) in comparison to ACs (1.40 ± 1.25). KRT19 levels also were highly incremented in cancer individuals (26.61 ± 45.46) related to healthy donors (1.21 ± 0.78). These differences were significant in both groups, yielding a *p*-value < 0.0001 for COL1A2 and a *p*-value = 0.0031 for KRT19 (Figure 2C).

**Figure 3:**
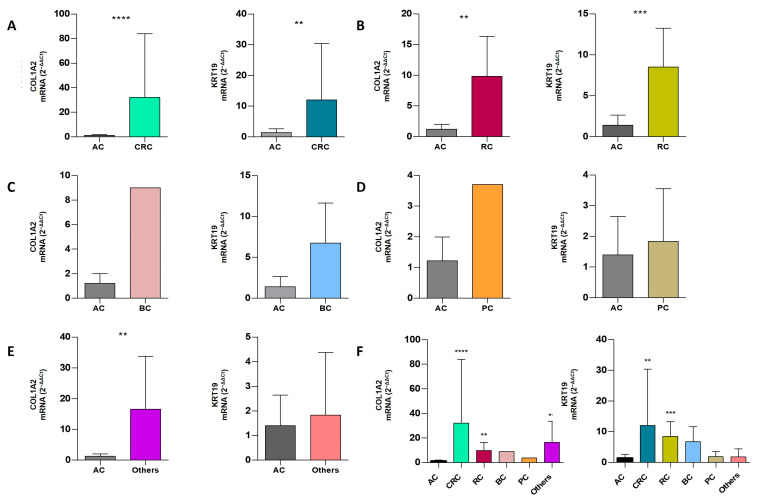
COL1A2/KRT19 mRNA fold change in the different group of tumors studied. Mann–Whitney U-test contrasts the mean in fold of control of two independent groups: AC (n = 13) vs. cancer patients’ groups (**A**) CRC (n = 31), (**B**) (RC; n = 6), (**C**) (BCC; n = 4), (**D**) (PC; n = 3), and (**E**) (Others E, n = 3) (gastric, mama and penile cancer). (**F**) Outlook for both markers and tumors. Graphs represent mean ±  SD, **** *p* < 0.0001, *** *p* < 0.001, ** *p* < 0.01, * *p* < 0.05.

Differences in relative gene expression were also analyzed for both biomarkers for the entire cohort and in the different cancer groups individually. Table 4 and Figure 3 show the obtained results. Expression levels of COL1A2 and KRT19 significantly increased in CRC and RC with respect to healthy donors (CRC: COL1A2, 32.11 ± 51.92, *p*-value < 0.0001; and KRT19, 12.06 ± 18.35, *p*-value = 0.0054; RC: COL1A2, 9.82 ± 6.50, *p*-value = 0.0088 and KRT19, 8.50 ± 4.75, *p*-value = 0.0008). In the group denominated Others, only COL1A2 was significantly increased (16.58 ± 17.14, *p*-value = 0.0044). However, intragroup variability observed in all groups was high. The bladder and prostate cancer groups seemed to have an increasing trend in both biomarkers, compared to ACs; nevertheless, the low number of cases in these groups did not allow us to draw conclusions for these types of tumors.

### 2.8. Combined Biomarker Prediction

**Figure 4:**
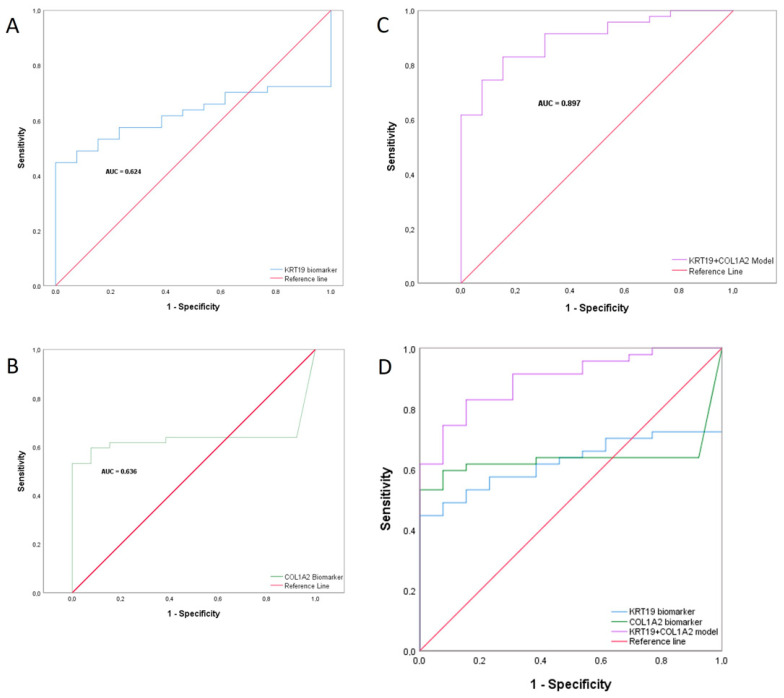
Graphical representation of the ROC curves for predicting cancer by profiling patients according to their expression level of KRT19, COL1A2 or both. (**A**) ROC curve for predicting cancer by KRT19 biomarker, AUC = 0.624 95% IC (0.490–0.757) Sensitivity = 0.617, Specificity = 0.615. (**B**) ROC curve for predicting cancer by COL1A2 biomarker, AUC = 0.636 95% IC (0.501–0.771) Sensitivity = 0.617, Specificity = 0.845. (**C**) ROC curve for predicting cancer using a combined biomarkers KRT19 + COL1A2, AUC = 0.897 95% IC (0.815–0.979), *p*-value = 0.000. Cut-off = 0.664. Sensitivity = 83%, Specificity = 84.6%. (**D**) COL1A2, KRT19 and combined model ROC curves. AUC denotes the area under the curve. The straight line in orange represents the line of discrimination (ND line), which divides the square of area 1.00 into two equal halves.

In order to design a diagnostic tool, we established an individual quantitative profile based on the level of biomarker expression, taking into account that not all patients express both markers. When the biomarkers (COL1A2 or KRT19) were not amplified, it was considered as no mRNA expression, and a value of 0 was assigned. As described above, we found significant differences in expression between the two groups (ACs vs. CPs) (Figure 2), and this quantitative expression profile was used as a tool in the statistical study and the establishment of the cut-off value. For this purpose, receiver operating characteristic (ROC) curve analysis was performed (Figure 4). 

ROC curves were constructed for each biomarker individually (KRT19, Figure 4A; COL1A2, Figure 4B) and for a combined model of both (Figure 4C). Figure 4D shows the differences between the three curves. We used both biomarkers to identify the performing combination of the data set using logistic regression, and the area under the curve (AUC) in the combined model was significantly higher than the individual biomarker model, as were the sensitivity and specificity (Figure 4C). The analysis of the combined model yielded an AUC of 0.897 (0.815–0.979). Moreover, it had an overall sensitivity of 0.830 and a specificity of 0.846 in the test (Table 5), compared to the individual markers COL1A2 [(AUC = 0.636 (0.501–0.771) S = 0.617, E = 0.845)] and KRT19 [ (AUC = 0.624 (0.490–0.757) S = 0.617, E = 0.615)] (Figure 4A and Figure 4B, respectively). 

**Figure 5:**
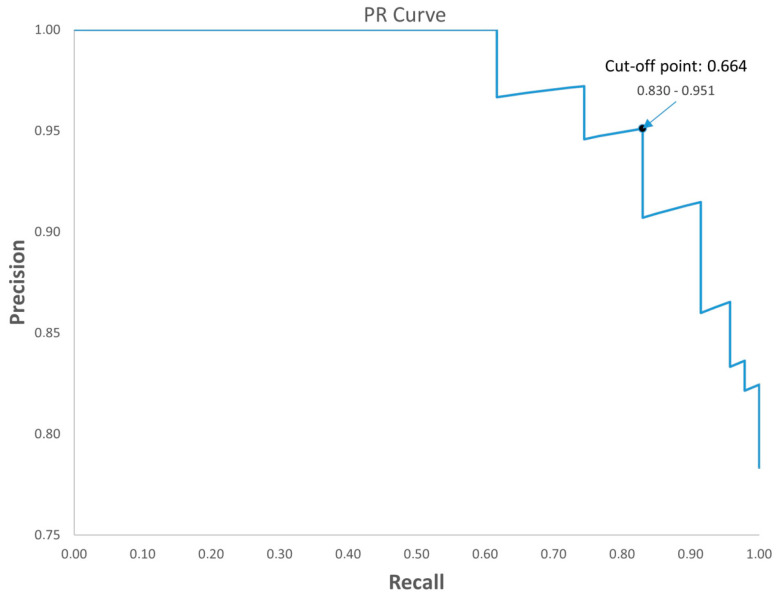
Graphical representation of the PR curve showing precision (0.830) and recall (0.951) values for the cut-off point (0.664).

Additionally, the positive predictive value (PPV) and negative predictive value (NPV) were 0.951 (0.839–0.986) and 0.579 (0.363–0.769), respectively. The diagnosis accuracy value was 0.833 (0.720–0.907), and to enhance the diagnostic strength of the test, we also calculated the likelihood ratios [18]. The positive likelihood ratio (PLHR) was 5.394, and the negative likelihood ratio (NLHR) value was 0.201 (Table 5). Regarding these data, we were also able to generate an algorithm and establish a cut-off value, which allowed us to discriminate based on the results obtained. This cut-off value was established as 0.664. In values lower than this cut-off value, asymptomatic patients were included, and in higher values, the CP group was included. To minimize the biases of possible unbalanced data, we constructed, in addition, a precision recall (PR) curve of the combined model (Figure 5). In the figure, we can observe the precision and recall values corresponding to the cut-off point. 

## 3. Discussion

Since the appearance of metastases is the leading cause of death in oncological patients, current efforts in early detection are more than justified [19]. In recent years, different challenges to be faced have been described when talking about early detection. These include the development of new models for understanding biology and prognosis, the determination of the risk of developing cancer, finding and validating cancer detection biomarkers, and the development of accurate technologies [6]. Initially, cancer research focused on the biology of the tumor cells themselves [20], but today, we know that the complexity of tumorigenesis goes beyond this, with bidirectional communication between specific cell types and the tumor microenvironment (TME) [21]. The objective of the present work was to develop a non-invasive screening method for the early detection of various carcinomas in plasma, using a panel that combines an epithelial and a stromal marker using RT-qPCR. This study was based on previous research in which we demonstrated that the analysis of RNA extracted from tumor-derived extracellular vesicles (TD-EVs) could be a correct approach to developing a diagnostic tool in oncological processes (16). Moreover, we observed an increased expression of COL1A2 and KRT19 in cancer patients with an advanced stage of the disease. Tumor and neoplastic stromal cells produce and release EVs loaded with macromolecules that facilitate tumor growth and dissemination, as well as therapeutic resistance [22]. In this context, EVs are being exploited as drug delivery strategies for cancer therapy and in the development of diagnostic biomarkers [23]. 

Multiple previous works have described the use of *KRT19* as biomarker in the differential diagnosis and characterization of most different epithelial tumors, and its role has been declared in many types of cancers, including colorectal [24], renal [25], bladder [26], gastric [27], breast [28,29], lung [14], pancreatic [30] and other [31,32] types of malignancies. In addition, its potential as a useful biomarker in the management of pathology is currently under investigation [16,33]. In our work, 85% of the cases had an increased expression of *KRT19*. This expression level was significantly increased in patients with colorectal and renal cancer. Otherwise, the low number of cases for bladder and prostate cancer did not allow for further conclusions, but positive trends could be observed, which led us to think that increasing the number of cases is key to determining the relationship between the biomarker and these types of tumors. 

As we have mentioned before, in recent years, there has been a new understanding providing evidence that ECM components should be explored as new diagnostic and prognostic biomarkers. *COL1A2* was included due to its involvement in disease progression and poor patient prognosis [34,35]. Previous studies have shown that *COL1A2* mRNA expression was significantly higher in malignant tissues than in premalignant and normal tissues. These reports have shown that the amount of type I collagen is increased in pancreas [36], colorectal [37], ovarian [38], breast [38,39], gastric [40] and lung cancer [41] and has also been described to play a role in metastasis, as exposure to type I collagen causes tumor cells to behave more invasively [42]. There is evidence that *COL1A2* promotes metastasis in breast cancer and contributes to poor survival in patients [34]. *COL1A2* has also been reported to be overexpressed in patients with bladder cancer, and its expression levels were positively associated with tumor grade [43], tumor size, and the depth of invasion [44]. Furthermore, the association between COL1A and chemoresistance in cancers has been demonstrated [35,45]. The understanding of ECM dynamics and the identification of its regulators will be key for the development of innovative therapeutic approaches [34]. 

The present work describes for the first time an increased combined expression of *COL1A2* and *KRT19* with respect to healthy participants. *COL1A2* was expressed, on average, 26.61-fold more in oncological patients than in the control group, and *KRT19* was expressed, on average, 9.68-fold more in the same way. These data showed that both biomarkers could have a potential role in finding and follow-up, enabling us to detect various types of cancer in the early stages. With our results, a non-invasive tool for early cancer detection was modeled combining the two biomarkers. Using liquid biopsy, we could detect patients with resectable lesions, establishing a cut-off value with an overall sensitivity of 0.830 and a specificity of 0.846. Recall and precision values were strong too for the cut-off point. The model showed a wide range of detection that includes early-stage cancers and an advanced colon adenoma (Tis). Therefore, the use of *COL1A2* in combination with the *KRT19* marker could contribute to the diagnosis of various types of cancer, regardless of stage. Moreover, our study is the first approximation of a non-invasive procedure for molecular diagnosis using a simple and affordable technology (qPCR multiplex), simultaneously identifying two types of biomarkers—one epithelial and the other mesenchymal. Thus, unlike other more complex and expensive existing methods designed with the same orientation, this procedure could be easily implemented in clinical practice.

However, our approach has encountered several limitations. It is necessary to increase the number of patients in both cohorts—asymptomatic controls and oncological subjects—to validate the technique. Moreover, we detected high intragroup heterogeneity, possibly due to this suboptimal number of patients in some cases. Another issue that could have influenced our results is that all the tumors were clinically detected, although most were detected in the early stages. Therefore, in order to overcome these drawbacks, the future of this project is based on, firstly, increasing the number of patients, as we need to improve the characterization of them all. Medical records must be very well-documented, and to reduce the great intragroup variability, we must increase the range of histological types, including adenocarcinomas, squamous carcinomas, etc., originating in different organs, focusing on the most prevalent and deadly tumors (lung, pancreas, colon, breast, prostate, etc.). This point would also help us to establish better sensitivity and specificity values, even specific for each type of tumor. On the other hand, the analysis at different points in the progress of the disease could allow us to correlate the values obtained with survival or recurrence, even for the detection of molecular complete remission or the existence of minimal residual disease. In a broader study, we could also study the expression of both biomarkers in primary tumors and try to correlate them with their plasma expression. Altogether, in order to achieve a prospective clinical application, COL1A2 and KRT19 might have a potential role as monitoring factors to screen early cancer, as well as prognostic factors predicting overall survival time, so this model could provide a new non-invasive option for the early detection and interception of tumors in population screening.

## 4. Materials and Methods

### 4.1. Patients and Human Samples

Blood samples were collected by the Biobank of A Coruña and analyzed in the Pathology Department at the University Hospital Complex of A Coruña. The Pathology laboratory is quality-certified [46]. Clinical data such as gender, age, tumor histology, and disease stage were obtained from the medical records. Patients belonged to a study approved by the Clinical Research Ethics Committee (approval registration number 2020/010), which was conducted in compliance with the Declaration of Helsinki. Written informed consent was provided, and custody and remnant sample storage was managed by the Biobank of A Coruña.

A total of 60 plasma samples belonging to 60 individuals were included in this study. Thirteen healthy ACs and forty-seven CPs undergoing curative resection underwent blood extraction for healthcare causes. Remnant samples were used for the study. 

### 4.2. Blood Sample Collection and RNA Isolation from Extracellular Vesicles

Peripheral whole blood of CPs was collected from each subject on the day of surgery in a 10 mL EDTA-K2 tube and processed after centrifugation within 4 h to avoid contamination with genomic DNA. AC peripheral whole blood was collected and processed equally in the same time interval. Samples were centrifuged at 2000× *g* for 20 min to collect 2 to 4 mL of plasma. The plasma obtained was passed through a 0.8 μm filter and stored at −80 °C. The processing of plasma samples and RNA isolation was carried out using the commercial ExoRNeasy Maxi Kit (QIAGEN, Hilden, Germany), and the manufacturer’s protocols were followed.

### 4.3. Multiplex RT-qPCR Assay

Genes of interest were co-amplified using the QIAGEN^®^ Multiplex PCR Kit (QIAGEN, Hilden, Germany) in a CFX96 C1000 Thermal Cycler (Bio-Rad Laboratories, Hercules, CA, USA) in a multiplex TaqMan assay. Compatible primer and probe sets that were specific for human gene targets were designed and synthesized by TIB Molbiol (Berlin, Germany). Multiplex PCR conditions were set according to the supplier: briefly, retrotranscription at 50 °C for 10 min, initial activation at 95 °C for 2 min and 40 cycles of 95 °C for 5 s and 62 °C for 30 s. PCRs were performed in 20 μL reaction volumes containing 7.8 μL H_2_O, 5 μL QuantiNova Multiplex PCR Master Mix, 1 μL of each primer-probe mix (20× primer-probe mix includes 16 µM forward/reverse primers and 5 µM TaqMan probe), 0.2 μL QuantiNova Multiplex RT mix, and 4 μL cDNA template. 

COL1A2 amplicons of 72 bp were amplified with PCR primers 5′-CCTGGACCAATGGGCTTA-3′ and 5′-GAAACCTTGAGGGCCTGG-3′; probes: 5′ hexachlorofluorescein (HEX) and 3′ BlackBerry™ Quencher 650 (BBQ-650™), sequence 5′-TAGAGGCCCACCTGGTGCAGC-3′. *KRT19* amplicons of 130 bp were amplified with PCR primers 5′-CAGCCACTACTACACGACCATC-3′ and 5′-CAAACTTGGTTCGGAAGTCATC-3′; probes: 5′ N-hydroxysuccinimide (LC610) and 3′ BBQ-650, sequence 5′-CAGCCAGACGGGCATTGTCG-3. *ACTB* amplicons of 99 bp were amplified with PCR primers 5′-CCACACTGTGCCCATCTACG -3′ and 5′-AGGATCTTCATGAGGTAGTCAGTCAG-3′; probes: 5′ tetramethylindo(di)-carbocyanines (CY5) and 3′ BBQ-650, sequence 5′-CAGGTCCAGACGCAGGATGGC-3’.

Relative gene expression was calculated using a modified comparative Ct method, (2−ΔΔCt), as described previously by Pfaffl [47]. Two replicates of each sample were analyzed for each gene. For the housekeeping gene (ACTB), a Ct ≤ 28 was considered positive. ACTB Ct values ≥ 29 were considered negative, and results were considered invalid. For epithelial and mesenchymal markers, a Ct value ≤ 39 with a sigmoidal curve was accepted as positive.

### 4.4. Amplification Efficiency (E)

To calculate the amplification efficiency (E) of the genes, a standard curve described above was used. The Ct line was adjusted to fit the standard curve with an acceptable R2 value (0.959–0.999). The amplification efficiency was calculated for each gene in singleplex and multiplex PCR, using the following equation: E = 100 × 10 (−1/slope).

### 4.5. Statistical Analysis

The nonparametric Mann–Whitney U-test was used for comparisons among groups. ΔCt and 2^−ΔΔCt^ were represented as mean ± SD, and unpaired t-tests (two-tailed) were used to test for differences between groups. A binary logistic regression model was used for analyzing data using IBM SPSS^®^ Statistics v27. For both biomarkers, a cut-off value was stablished using Youden’s J index (value combining highest sensitivity and specificity), through ROC curve analysis, and AUCs were calculated. Additionally, a PR curve was produced in order to prove the efficiency of the approach. When more than one value fulfilled this condition, the cut-off value allowing for higher sensitivity was chosen. Accordingly, the sensitivity, specificity, diagnostic accuracy, PLHR, NLHR, PPV and NPV were calculated. Confidence intervals (CIs) were calculated by the Wilson Score Interval. The statistical significance was determined at α-limit = 5% in all analyses. 

## 5. Patents

B.O.A., L.S.E-P., M.O.A., S.D.H., L.S. and Á.C. filed a patent application that details the potential role of the plasmatic TD-EV mRNA of KRT19 and COL1A2 as a non-invasive diagnostic tool in the diagnosis of several neoplastic processes.

## Figures and Tables

**Table 1:** Reproducibility of intra- and inter-assay with different dilution of human XpressRef Universal Total ARN (Qiagen) by multiplex RT-qPCR. Coefficient of variation (CV).

Dilutions	ARN (ng)	Ct Values for the Different Dilutions of Human XpressRef Universal Total ARN					
		β-Actin Mean Ct ± SD	CV (%)	KRT19 Mean Ct ± SD	CV (%)	COL1A2 Mean Ct ± SD	CV (%)
Intra-assay	200	17.02 ± 0.44	2.58	19.83 ± 0.23	1.18	17.91 ± 0.11	0.63
	20	19.82 ± 0.44	2.21	22.50 ± 0.22	0.96	21.32 ± 0.37	1.75
	2	23.45 ± 0.16	0.68	26.65 ± 0.26	0.99	24.80 ± 0.38	1.55
	0.2	27.11 ± 0.29	1.07	29.61 ± 0.33	1.12	28.44 ± 0.24	0.85
	0.02	30.19 ± 0.23	0.77	33.32 ± 0.17	0.51	31.78 ± 0.27	0.85
	0.002	33.13 ± 0.23	0.69	36.50 ± 1.15	3.16	35.31 ± 1.05	3.00
	0.0002	36.61 ± 0.31	0.84	-	-	-	-
Inter-assay	200	16.89 ± 0.39	2.33	19.97 ± 0.29	1.45	19.22 ± 0.68	3.52
	20	20.04 ± 0.44	2.17	23.30 ± 0.39	1.66	22.65 ± 0.68	3.01
	2	23.29 ± 0.26	1.10	26.63 ± 0.31	1.15	25.70 ± 0.88	3.42
	0.2	27.15 ± 0.35	1.30	30.16 ± 0.28	0.93	29.68 ± 0.67	2.24
	0.02	30.89 ± 0.23	0.74	33.96 ± 0.61	1.81	33.38 ± 0.81	2.44
	0.002	33.45 ± 0.43	1.27	36.68 ± 0.92	2.50	35.77 ± 0.95	2.66
	0.0002	36.63 ± 0.57	1.54	-	-	-	-

**Table 2:** Efficiency and degree of linearity of singleplex and multiplex PCRs with the three gene targets.

Target	Multiplex	Slope	R2	Efficiency
COL1A2	Singleplex	−3.325	0.987	99.9%
KRT19	Singleplex	−3.240	0.996	103.5%
ACTB	Singleplex	−3.453	0.999	94.8%
COL1A2	Triplex	−3.043	0.957	113.1%
KRT19	Triplex	−3.594	0.979	89.8%
ACTB	Triplex	−3.330	0.995	99.7%

**Table 3:** Demographical and clinical-pathological characteristics of the population included in the study.

Clinical and Pathological Features of Individuals Included in This Study (n = 60)				
**Clinic Pathological Features**			**AC (n = 13)**	**CPs (n = 47)**
Age Median (Range)			41.46 (26–58)	70.36 (46–90)
Gender				
Female			9 (69.23%)	24 (51.06%)
Male			4 (30.77%)	23 (48.94%)
Curative Resections			-	47 (100%)
**Clinical and Pathological Features of Patients with Tumor Process (n = 47)**				
**Colorectal Cancer (CRC) (n = 31)**			**Prostate Cancer (PC) (n = 3)**	
Age mean (range)		71.45 (40–90)	Age mean (range)	71.66 (64–86)
Histological Type			Histological Type	
Adenocarcinoma		31	Adenocarcinoma	3
Clinical stage			Clinical stage	
Tis */I/II		20	II	2
III		11	III **	1
**Renal Cancer (RC) (n = 6)**			**Bladder Cancer (BC) (n = 4)**	
Age mean (range)		61.66 (44–79)	Age mean (range)	72.00 (58–86)
Histological Type			Histological Type	
CCRCC		6	Urothelial	4
Clinical stage			Clinical stage	
I/II		3	0a	1
III		3	II	3
**Others (n = 3)**				
	**Age**	**Sex**	**Histological Type**	**Clinical Stage**
Leiomyosarcoma	74	female	Renal leiomyosarcoma	III
Breast cancer	74	female	Ductal invasive	I
Penile cancer	66	male	Squamous	II

* Tis adenocarcinoma: lamina propria infiltration; ** III stage: pT2N0 patient with > 20 plasmatic PSA level.

**Table 4:** Amplification percentages of each tumoral biomarker. Mean ± SD and *p*-values were from two-tailed Mann–Whitney test. *p*-values shown in red were statistically significant. The statistical significance was determined at α-limit = 5% in all analysis. ACs (asymptomatic controls); CPs (cancer patients); CRC (colorectal cancer); RC (renal cancer); BC (bladder cancer); PC (prostate cancer); nd (not determined).

Groups	N	Amplification			Mean ± SD		*p*-Value	
		COL1A2	KRT19	COL1A2 and KRT19	COL1A2	KRT19	COL1A2	KRT19
AC	13	12 (92.30%)	13 (100%)	12 (92.30%)	1.21 ± 0.78	1.40 ± 1.25	-	-
CP	47	30 (63.83%)	38 (80.85%)	21 (44.68%)	26.61 ± 45.46	9.69 ± 15.07	0.0001	0.0031
CRC	31	22 (70.96%)	24 (77.42%)	15 (48.39%)	32.11 ± 51.92	12.06 ± 18.35	0.0001	0.0054
RC	6	3 (50%)	5 (83.33%)	1 (16.67%)	9.82 ± 6.50	8.50 ± 4.75	0.0088	0.0008
BC	4	1 (25%)	4 (100%)	1 (25%)	nd	6.74 ± 4.89	nd	0.1567
PC	3	1 (33.33%)	3 (100%)	1 (33.33%)	nd	1.84 ± 1.71	nd	0.5054
Others	3	3 (100%)	2 (66.67%)	2 (66.67%)	16.58 ± 17.14	nd	0.0044	nd
Total	60	42 (70%)	51 (85%)	33 (55%)	nd	nd	nd	nd

**Table 5:** Diagnostic accuracy of both biomarkers combined.

Sensitivity	0.830	(0.699–0.911)
Specificity	0.846	(0.578–0.957)
Positive Predictive Value	0.951	(0.839–0.986)
Negative Predictive Value	0.579	(0.363–0.769)
Diagnostic Accuracy	0.833	(0.720–0.907)
Positive Likelihood Ratio	5.394	(2.004–14.520)
Negative Likelihood Ratio	0.201	(0.152–0.265)

## Data Availability

The data presented in this study are available in the article. Any other related-information or document does not present in this study are available on request.

## Supplementary Materials

The following supporting information can be downloaded at: https://www.mdpi.com/article/10.3390/ijms25179567/s1.
