# Supplementary material for: Simultaneous Detection of Collagen I Alpha II and Cytokeratin 19 mRNA by Multiplex qPCR in Liquid Biopsy in Diagnosis of Patients with Resectable Solid Tumors
Source: Int J Mol Sci. 2024 Sep 3;25(17):9567. doi: 10.3390/ijms25179567 (PMC11395584; doi:10.3390/ijms25179567)
Supplement: Supplementary file 1 [file ijms-25-09567-s001.zip › ijms-3128384-supplementary.pdf]

ID	Age	Status	Sex	Resec	ΔCt		2^-ΔΔCt		model predicted probability
		AC (0) CP (1)	M (0) F (1)	No resec (0) CRC (1) UC (2) RC (3) PC (4) Others (5)	COL1A2	*KRT19*	*COL1A2*	*KRT19*	*cut off* 0.664
AC1	55	0	0	0	11,79	10,82	0,562	1,789	0,477
AC2	33	0	0	0	11,24	13,505	0,823	0,278	0,316
AC3	52	0	1	0	11,42	9,55	0,726	4,315	0,801
AC4	52	0	1	0	12,942	12,219	0,253	0,679	0,293
AC5	43	0	1	0	10,741	12,446	1,163	0,580	0,398
AC6	38	0	1	0	10,513	12,649	1,362	0,504	0,415
AC7	33	0	1	0	10,236	10,633	1,651	2,037	0,661
AC8	27	0	1	0	9,852	11,086	2,153	1,488	0,657
AC9	50	0	0	0	11,025	11,325	0,955	1,260	0,461
AC10	58	0	1	0	10,52	11,95	1,355	0,818	0,457
AC11	42	0	1	0		12,666	0,000	0,498	0,246
AC12	30	0	0	0	9,36	9,86	3,029	3,480	0,904
AC13	26	0	1	0	11,865	12,863	0,533	0,434	0,299
CP1	46	1	0	1	4,16	8,065	111,329	12,078	1,000
CP2	67	1	1	1	10,562	12,235	1,317	0,671	0,432
CP3	81	1	1	1	7,055	9,049	14,966	6,106	1,000
CP4	73	1	1	1	9,649	10,12	2,480	2,906	0,834
CP5	82	1	1	1		7,218	0,000	21,729	1,000
CP6	55	1	0	1		5,375	0,000	77,933	1,000
CP7	90	1	0	1	8,338		6,149	0,000	0,892
CP8	83	1	1	1		6,607	0,000	33,172	1,000
CP9	65	1	1	1	4,795	12,666	71,696	0,498	1,000
CP10	82	1	1	1	7,79	8,927	8,994	6,645	0,999
CP11	79	1	0	1	3,8	8,438	142,895	9,328	1,000
CP12	72	1	0	1	7,18	10,767	13,722	1,856	0,999
CP13	77	1	1	1		6,835	0,000	28,327	1,000
CP14	75	1	0	1	9,046	8,11	3,765	11,710	0,999
CP15	73	1	0	1	7,838		8,697	0,000	0,973
CP16	73	1	0	1	9,712		2,373	0,000	0,490
CP17	77	1	1	1	6,914	14,321	16,507	0,158	1,000
CP18	89	1	1	1	9,95	12,25	2,012	0,664	0,529
CP19	56	1	0	1		8,59	0,000	8,394	0,961
CP20	53	1	1	1		11,18	0,000	1,394	0,348
CP21	67	1	1	1	7,62		10,117	0,000	0,988
CP22	74	1	0	1	8,937	11,315	4,060	1,269	0,835
CP23	40	1	1	1	5,419	6,123	46,508	46,399	1,000
CP24	58	1	1	1	3,361	9,158	193,752	5,663	1,000
CP25	80	1	1	1	8,686		4,831	0,000	0,796
CP26	81	1	0	1		9,074	0,000	6,003	0,870
CP27	75	1	1	1		10,665	0,000	1,992	0,425
CP28	88	1	0	1	7,41		11,698	0,000	0,995
CP29	63	1	0	1		9,52	0,000	4,405	0,736
CP30	71	1	0	1	7,66		9,840	0,000	0,986
CP31	70	1	0	1	6,731	15,157	18,740	0,089	1,000
CP32	86	1	1	2		8,874	0,000	6,895	0,916
CP33	75	1	0	2		8,72	0,000	7,670	0,944
CP34	58	1	0	2		8,06	0,000	12,120	0,995
CP35	69	1	1	2	7,79	13,54	8,992	0,272	0,980
CP36	79	1	1	3	6,97	7,81	15,875	14,413	1,000
CP37	48	1	0	3		9,03	0,000	6,187	0,881
CP38	44	1	0	3		8,99	0,000	6,361	0,891

CP39	67	1	1	3	7,55		10,620	0,000	0,991
CP40	68	1	1	3		10,04	0,000	3,072	0,573
CP41	64	1	1	3	9,39	8,02	2,966	12,461	0,999
CP42	79	1	1	5	5,79		35,969	0,000	1,000
CP43	76	1	0	4	9,07	12,19	3,703	0,692	0,750
CP44	64	1	0	4		9,73	0,000	3,809	0,668
CP45	75	1	0	4		11,64	0,000	1,013	0,302
CP46	74	1	1	5	9,178	16,741	3,436	0,030	0,642
CP47	66	1	0	5	7,59	9,8	10,329	3,628	0,998
